# Development and validation of the Arabic children’s strong communication scale: a pilot study

**DOI:** 10.3389/fpsyg.2024.1380296

**Published:** 2024-09-27

**Authors:** Munassir Alhamami, Ahmed Alduais, Muhammad Alasmari, Fawaz Qasem

**Affiliations:** ^1^Department of English, King Khalid University, Abha, Saudi Arabia; ^2^Department of Human Sciences (Psychology), University of Verona, Verona, Italy; ^3^Department of English Language and Literature, College of Letters and Arts, University of Bisha, Bisha, Saudi Arabia

**Keywords:** Arabic children’s strong communication scale, Children’s communication checklist-2, communication skills, pragmatic language, cultural adaptation

## Abstract

**Background:**

The assessment of communication skills in Arabic-speaking children has been challenged by a lack of culturally and linguistically appropriate tools. The Arabic children’s strong communication scale (ACSCS) was developed in response to this need, adapting the children’s communication checklist-2 (CCC-2) to better suit the Arabic context.

**Aims:**

This study aimed to validate the ACSCS and establish its utility in measuring communication strengths among Arabic-speaking children.

**Methods:**

A total of 102 participants completed the questionnaire, which included relatives, teachers, and speech therapists of children aged 4–16. Twenty-two items were developed that targeted children communication strengths. The items were based on a theoretical framework that encompassed language proficiency, social communication, and behavioral aspects. Exploratory factor analysis (EFA) and confirmatory composite analysis (CCA) were employed to validate the structure of the ACSCS. The scale’s reliability was tested using Cronbach’s alpha and composite reliability, while its validity was assessed through convergent and discriminant validity measures.

**Results:**

The EFA and CCA confirmed a clear distinction of communication strengths. Reliability analyses indicated high internal consistency for the ACSCS. Discriminant validity was established, showing that the ACSCS accurately measures distinct facets of communication skills.

**Conclusion:**

The ACSCS is a valid and reliable instrument for assessing communication skills in Arabic-speaking children, reflecting strengths across various domains.

**Implications:**

The scale has significant implications for educational and clinical settings, providing a culturally sensitive tool for practitioners and researchers to assess and support the communication development of Arabic-speaking children.

## Introduction

### Uses of CCC and CCC-2

The Children’s communication checklist (CCC), initially developed by Bishop (1998), was designed to assess communicative impairments not adequately evaluated by standardized language tests. It included pragmatic abnormalities seen in social communication. Bishop and Baird (2001) expanded on its use, demonstrating its effectiveness in a clinical setting, particularly in differentiating children with pervasive developmental disorders from those with specific learning disabilities. Nathan (2002) further substantiated its utility by highlighting its effectiveness in identifying functional communication problems in children with speech difficulties. Botting (2004) and Geurts et al. (2004) reinforced the CCC’s usefulness in clinical settings and research for discerning pragmatic deficits in children with attention deficit hyperactivity disorder and high functioning autism.

Norbury et al. (2004) introduced the children’s communication checklist 2nd edition (CCC-2), which improved upon the original CCC by providing a more robust screening for communication disorder and identifying pragmatic/social interaction deficits. Also, Britton (2005) found the CCC-2 to be complementary to clinician assessment and particularly valuable in community pediatric clinics. Subsequent studies (e.g., Bishop et al., 2006; Verté et al., 2006) utilized the CCC-2 to explore the broader phenotype in autism and differentiate autism spectrum subtypes, highlighting the scale’s discriminative power. Other researchers such as Eadie (2007) and Sarimski (2006) noted the CCC-2’s reliability and validity in clinical assessment of children with intellectual disabilities and its potential in psychiatric settings, respectively.

### Validation of CCC and CCC-2

Helland and Heimann (2007) demonstrated the CCC’s effectiveness in a Norwegian sample, while Helland et al. (2009) provided evidence for the CCC-2’s reliability and sensitivity in identifying children with language impairment. Further, Geurts et al. (2009) and Ketelaars et al. (2009) supported the CCC’s construct validity and screening potential for pragmatic language impairment. Besides, Yliherva et al. (2009) found the CCC and CCC-2 effective in evaluating communication skills in children with Fragile X syndrome. Volden and Phillips (2010) compared the CCC-2 with the test of pragmatic language, illustrating the CCC-2’s superior ability to identify pragmatic language impairment in children with autism spectrum disorders. Further cross-cultural adaptations and validations were conducted by (e.g., Chuthapisith et al., 2014; Costa et al., 2013; Crespo Eguílaz et al., 2016; Glumbić and Brojčin, 2012; Hoffmann et al., 2013; Mahmoodi et al., 2014; Vézina et al., 2013), confirming the CCC-2’s reliability and validity in various languages and cultures. The Cronbach’s alpha values reported in the literature for the CCC and CCC-2 demonstrate good internal consistency (e.g., Bishop, 1998; Norbury et al., 2004). We will provide specific Cronbach’s alpha values for ACSCS in the results section. The ACSCS builds upon the strengths of the CCC-2, a widely used and validated tool for assessing pragmatic language skills in children. The CCC-2’s structure and focus on pragmatic communication make it a suitable foundation for the ACSCS. However, we have adapted the items and context to reflect the unique features of the Arabic language and culture. For instance, items that refer to specific cultural references or social situations common in Western societies will be replaced with those relevant to Arabic-speaking communities.

### Measuring children’s communication skills

Several studies including (Chuthapisith et al., 2014; Mahmoodi et al., 2014; Timler, 2014; Vaïsänen et al., 2014) explored the CCC-2’s diagnostic accuracy and its association with various aspects of communication, including structural language, pragmatics, and social cognition. Other studies such as Crespo Eguílaz et al. (2016) and Song et al. (2016) highlighted the CCC-2’s ability to identify disorders of pragmatic use of language and differentiate clinical subtypes. Further, Tanaka et al. (2017) utilized cluster analysis to reveal communication impairment subtypes within Japanese children with autism spectrum disorders, while McGownd (2018) investigated CCC-2 measurements in internationally adopted children.

Hammond (2019) examined the agreement between parent and teacher ratings on the CCC-2, and Lane et al. (2019) provided insights into communication abilities in children with Sotos syndrome. Ferrara et al. (2020) and Andrés-Roqueta et al. (2021) underscored the CCC-2’s capacity to differentiate autism spectrum disorders from other neurodevelopmental disorders and its association with executive functions. Most recently, a number of studies (e.g., Aghaz et al., 2022; De La Torre Carril et al., 2021; Fisher et al., 2022; Girimaji et al., 2023; Nowell et al., 2022) confirmed the CCC-2’s clinical utility in various settings, including traumatic brain injury and attention deficit hyperactivity disorder, and its psychometric robustness in diverse populations.

The CCC and CCC-2 have been extensively validated and adapted across cultures, demonstrating their utility in identifying pragmatic language impairments and differentiating between various developmental disorders. The tools have proven effective in both clinical and research settings, offering a robust measure for assessing communication competencies. While direct assessments remain crucial, the CCC-2 complements these measures by providing insights into a child’s communicative abilities across different contexts, as reported by parents and teachers. Despite the strengths of the CCC and CCC-2, limitations include the reliance on subjective parental reports and potential cultural biases in translation. Future research should focus on larger, more diverse samples and the development of normative data across different languages and cultures. Longitudinal studies would provide further insight into the developmental trajectory of communication skills in children with various neurodevelopmental disorders.

In summary, the CCC and CCC-2 are invaluable tools for assessing children’s communication skills, offering a pragmatic language perspective that is often overlooked in traditional language assessments. The extensive research across various populations underscores their importance and establishes them as critical instruments in the field of child language development and disorders.

### Rationale for the study

The endeavor to develop the ACSCS is underpinned by the considerable insights gleaned from the extensive application and evaluation of the CCC and its second edition (CCC-2) across various languages and cultures. The CCC and CCC-2 have been instrumental in identifying communication impairments, particularly in the realm of pragmatic language skills, which are crucial for effective social interaction. However, the global research on CCC and CCC-2 also highlights the pressing need for culturally sensitive tools that align with the linguistic nuances and cultural contexts of specific populations, such as Arabic-speaking children.

The ACSCS aims to fill this void by providing a culturally adapted tool that is sensitive to the unique communicative nuances of the Arabic language and its dialects. The scale’s development is informed by a growing body of evidence that underscores the importance of culturally and linguistically appropriate assessment tools. By considering the specific social and linguistic features of Arabic-speaking communities, the ACSCS will enhance the accuracy of communication assessments and support the identification of communication competencies and difficulties among Arabic-speaking children.

Moreover, the ACSCS is designed to integrate seamlessly into educational and psychological frameworks, offering a valuable resource for professionals in these fields. By facilitating the early detection and intervention of communication challenges, the ACSCS will contribute to the educational success and psychological well-being of children. It will also empower parents and guardians with a deeper understanding of their children’s communicative abilities, thereby supporting home-based language development strategies.

Briefly, the ACSCS is not merely an adaptation of existing tools like the CCC and CCC-2, but an innovative advancement tailored for the Arabic-speaking context. Its development is a testament to the necessity of culturally informed research and the creation of assessment instruments that respect and reflect the linguistic diversity of children worldwide. Through its implementation, the ACSCS will not only enrich the current landscape of communication assessment but also pave the way for future research and policy developments that champion the linguistic rights and needs of children in the Arabic-speaking world.

## Method

### Sample

In this study, 121 participants initially filled out the questionnaire. However, 19 were excluded due to incomplete responses, leaving 102 participants whose data were fully available and included in the final analysis. Among these participants, the majority (69%, or 83 participants) were brothers and sisters of the children involved in the study. There were also parents who answered: 10 fathers and 12 mothers, making up about 8 and 10% of the answers, respectively. Seven teachers and nine speech therapists also filled out the questionnaire, which is about 6 and 7% of the total answers. This shows that people with different connections to the children shared their views in the study.

The study looked at children aged between 4 and 16 years old. The age that was most common was 7 years old, making up 14% of the study. The ages 14 to 16 were the least common, each age group making up about 2.5% of the total. The ages 4, 5, and 8 were also a big part of the study, each group being more than 11% of the participants who took part. Mostly, the study focused on younger children, with 63% of the participants talking about children aged between 4 and 8 years.

The study also looked at whether the children were boys or girls based on what their relatives (like fathers, sisters, or speech therapists) said. There were more boys in the study, with 80 boys making up 66% of the total. There were 41 girls, which is about 34% of the study. This shows that there were more boys than girls in the study. Table 1 summarizes the main demographic characteristics of the participants.

**Table 1:** Demographic characteristics and participation.

Relationship to children	
Brothers and sisters	83 (69% of total)
Fathers	10 (approx. 8% of total)
Mothers	12 (approx. 10% of total)
Teachers	7 (approx. 6% of total)
Speech therapists	9 (approx. 7% of total)
Gender distribution of children	
Boys	80 (66% of total children)
Girls	41 (34% of total children)

### Instrument

#### Theoretical framework model for measuring ACSCS

We used previous published instruments that measure children strong communication skills to build the theoretical framework (e.g., Bishop, 2003, 2021). The theoretical framework model focuses on measuring aspects of strong communication skills in children, the model is structured to highlight positive language abilities, social communication strengths, and adaptive behavior patterns.

1. Strong specific language aspects

- A. Speech proficiency: emphasizes clear and articulate sound production and word articulation.
- B. Syntax mastery: involves adept use of sentence structures and grammatical accuracy.
- C. Semantic competence: pertains to the rich understanding and usage of the meanings of words and phrases.
- D. Coherent discourse: focuses on the ability to construct clear, logical, and well-structured discourse.

2. Strong social communication aspects

- E. Appropriate initiation: demonstrates skill in beginning communication in a socially appropriate and contextually sensitive manner.
- F. Varied language use: reflects the ability to use language flexibly and creatively, avoiding repetitive or rigid language patterns.
- G. Contextual language proficiency: involves the adept use of language in various social contexts, indicating a high level of pragmatics.
- H. Nonverbal communication skills: highlights strengths in both using and interpreting nonverbal cues such as body language, facial expressions, and gestures.

3. Strong behavioral aspects

- I. Social interaction prowess: showcases strengths in actively engaging, responding, and adapting in social interactions and settings.
- J. Diverse personal interests: indicates a well-rounded, diverse range of interests and activities, reflecting flexibility and openness to new experiences.

The model has several applications, allowing for a comprehensive evaluation of a child’s communication skills by considering linguistic abilities, social aptitude, and behavioral adaptability. The model acknowledges that proficiency in specific language aspects can enhance social communication skills, and both can positively influence a child’s behavior in social settings. By assessing children across these constructs, speech therapists, educators and practitioners can identify areas of strength as well as those needing further development. This theoretical framework provides a structured approach to understand and measure the various dimensions of strong communication skills in children. It can be instrumental in guiding educational strategies, language therapy interventions, and research in child development and education.

We developed an Arabic scale to measure ACSCS by adapting specific items from Bishop’s CCC2 (2003). The scale consists of 20 items organized into ten lower constructs. These constructs are grouped into three higher constructs. A. Speech proficiency, B. Syntax mastery, C. Semantic competence, and D. Coherent discourse measure strong specific language aspects. E. Appropriate initiation, F. Varied language use, G. Contextual language proficiency, and H. Nonverbal communication skills focus on strong social communication aspects. Finally, I. Social interaction prowess and J. Diverse personal interests assess strong behavioral aspects, as outlined in the theoretical framework above.

Initially, we developed 70 items, 50 items that measure children weakness and reported in another study and 20 items that are reported in this study. Each item is measured using a 4-point Likert scale. The scale options are:

1. Less than once a week (or never).
2. At least once a week, but not every day.
3. Once or twice a day.
4. Several times (more than twice) a day (or always).

To enhance reliability, items from different constructs were interleaved throughout the questionnaire items. For more information about the ACSCS items, see Appendix A.

### Design

The ACSCS study adopted a cross-sectional design to validate the newly adapted assessment tool within the Arabic-speaking pediatric population. This design facilitated the collection of data at a single point in time, allowing for the efficient examination of the scale’s psychometric properties across a diverse sample. The questionnaire was disseminated electronically via Google Forms, ensuring wide accessibility and convenience for participants. The design incorporated several statistical tests to rigorously evaluate the scale’s construct validity and factor structure. Additionally, reliability was assessed through internal consistency metrics, while the scale’s ability to discriminate between communication competencies and deficits was scrutinized using discriminant validity tests. This methodological approach ensured a comprehensive validation process, establishing the ACSCS as a reliable and valid instrument for assessing communicative abilities in Arabic-speaking children.

### Procedure

#### Content validity and cross-cultural validation

The research commenced following the university’s Institutional Review Board approval (ECM#2023-2007), focusing on the content validity and cross-cultural adaptability of the questionnaire items. To accurately measure participants’ experiences, the researchers embarked on a detailed process of adapting the questionnaire to the Arabic language and cultural context, which encompassed several critical steps. Two researchers, both proficient in native Arabic, undertook the translation of the questionnaire. Their goal was not only to ensure linguistic fluency but also to maintain the accuracy of the questionnaire’s content. Beyond simple translation, the team dedicated effort to modify items to better resonate with Arabic speakers. This included integrating nuances of the Arabic language, phonetic considerations, and cultural references that would be more relevant and meaningful to the target audience. The translated items were then presented to five Arabic speakers who had no proficiency in English. Each of these reviewers, holding university degrees ranging from PhDs in Arabic studies to BAs in Islamic Culture from Saudi universities, assessed the items for clarity, understanding, and cultural appropriateness. Following the comprehension review, the researchers engaged in discussions with the reviewers to gather their feedback. This feedback was meticulously incorporated into the questionnaire, refining and enhancing its relevance and clarity.

Once the modifications were made, the revised questionnaire was formatted into a Google Form and presented to the entire research team. This step was crucial for a final review, ensuring the integrity and accuracy of the questionnaire items. After reaching a consensus on the questionnaire’s content among all researchers, it was distributed through both personal and professional networks. This strategy was employed to ensure a wide and relevant reach, encompassing friends and relatives with children, as well as professional speech therapists. These thorough procedures not only facilitated the achievement of content validity and cross-cultural adaptation of the questionnaire but also ensured a comprehensive measurement of the participants’ experiences.

### Statistical analysis

To ensure a rigorous assessment of the data collected through the ACSCS, a comprehensive statistical analysis was conducted. Initially, exploratory factor analysis (EFA) was employed to identify the underlying factor structure of the questionnaire items, helping to confirm the dimensions of communication competencies as hypothesized in the study design. This was followed by confirmatory composite analysis (CCA) to verify the factor structure suggested by the EFA and to assess the fit of the model to the observed data, providing a measure of how well the proposed model represented the data collected (Kline, 2023).

The use of both EFA and CFA was essential in the current study. Despite having a theoretical basis for the domains of ACSCS, EFA was conducted initially to explore the underlying factor structure of this newly developed scale. Since the ACSCS represents a novel adaptation for the Arabic-speaking context, it was crucial to employ EFA to identify potential dimensions and verify whether the data conformed to our theoretical expectations. This exploratory step allowed us to refine the scale by examining factor loadings and ensuring that each item contributed meaningfully to the constructs being measured. Following the EFA, CFA was then utilized to confirm the factor structure identified in the exploratory phase. This step was necessary to validate the proposed model and ensure that the identified factor structure fit the data adequately. By employing both EFA and CFA, we were able to establish both the empirical validity and the theoretical robustness of the ACSCS, thus providing a reliable and culturally sensitive tool for assessing communication skills among Arabic-speaking children (Hair et al., 2019; Willaby et al., 2015).

For the assessment of reliability, Cronbach’s alpha was calculated for each scale to determine the internal consistency and to ensure that similar items on the same scale effectively measured the same construct. Composite reliability was also computed to affirm the consistency of the scales. In terms of validity, convergent validity was examined by assessing the average variance extracted (AVE) for each factor, ensuring that a significant amount of the variance in the items was explained by the factors. Discriminant validity was evaluated by comparing the square root of the AVE with the inter-correlations among factors, confirming that each factor is distinct and not overly correlated with other factors. The researchers employed PLS-SEM using SmartPLS application Version 4.0.9.8 (Ringle et al., 2024) to analyze the data.

## Results

This section offers a detailed examination of the psychometric properties of the ACSCS. It begins with an EFA that scrutinizes the factor structure of the questionnaire, ensuring that the items align with their respective constructs, which are divided into two domains: communication weaknesses and strengths. The efficacy of the items in capturing the intended constructs is reflected in the factor loadings and their uniqueness. This section then transitions into CCA, employing partial least squares structural equation modeling (PLS-SEM) to confirm the measurement model’s performance. The model’s reliability is assessed using Cronbach’s alpha and composite reliability, while convergent and discriminant validity measures underpin the validity assessments. The results embrace both individual item and construct level analyses, providing a comprehensive evaluation of the internal consistency, reliability, and construct validity of the ACSCS. Additionally, the discriminant validity analysis ensures that each construct measures unique aspects of communication skills, while the correlation matrix elucidates the interrelationships among the constructs. The higher-order construct’s evaluation within the structural model uses path coefficients, effect size, and coefficient of determination to affirm the ACSCS’s predictive capacity. Collectively, these analyses establish the ACSCS as a robust tool for assessing communication skills in Arabic-speaking children.

### Exploratory factor analysis

The EFA was conducted to ensure that the questionnaire items appropriately loaded onto their respective factors. The questionnaire comprised 70 items, divided into two sections: 50 items assessing children’s weaknesses in communication across ten constructs (5 items per construct), and 20 items evaluating children’s strengths in communication (2 items for each of the ten constructs). The mean score for each construct was calculated, encompassing a total of 20 constructs - ten for weaknesses and ten for strengths. EFA was then applied to these mean scores. Table 2 presents the results of the analysis. In assessing the practical and statistical significance of factor loadings, a threshold of +0.40 was set as the benchmark for significant importance.

**Table 2:** Exploratory factor analysis results.

Factor loadings			
Factor	1	2	Uniqueness
A. Weak speech proficiency	0.766		0.485
A. Strong speech proficiency		0.743	0.422
B. Weak syntax mastery	0.828		0.28
B. Strong syntax mastery		0.764	0.392
C. Weak semantic competence	0.933		0.223
C. Strong semantic competence		0.571	0.56
D. Weak coherent discourse	0.864		0.227
D. Strong coherent discourse		0.865	0.33
E. Weak appropriate initiation	0.567		0.454
E. Strong appropriate initiation		0.528	0.636
F. Weak varied language use	0.77		0.361
F. Strong varied language use		0.868	0.298
G. Weak contextual language proficiency	0.846		0.245
G. Strong contextual language proficiency		0.503	0.52
H. Weak nonverbal communication skills	0.75		0.38
H. Strong nonverbal communication skills		0.762	0.421
I. Weak social interaction prowess	0.814		0.359
I. Strong social interaction prowess		0.792	0.368
J. Weak diverse personal interests	0.509		0.481
J. Strong diverse personal interests		0.741	0.377

“Minimum residual” extraction method was used in combination with a “oblimin” rotation.

**Factor 1**: this factor is primarily associated with constructs measuring children’s communication weaknesses. It includes items related to speech proficiency, syntax mastery, semantic competence, coherent discourse, and nonverbal communication skills, among others. The results were reported in another study using different theoretical framework and statistical analysis procedures.

**Factor 2**: this factor is primarily associated with constructs measuring children’s communication strengths. It includes items related to speech proficiency, syntax mastery, varied language use, contextual language proficiency, nonverbal communication skills, social interaction prowess, and diverse personal interests. The results are reported in the current study.

The EFA results suggest that the questionnaire items generally align with their intended constructs, demonstrating a clear distinction between measures of communication weaknesses and strengths. These findings support the validity of the questionnaire’s structure and its potential for accurately assessing children’s communication skills.

### Confirmatory composite analysis

**Figure 1:**
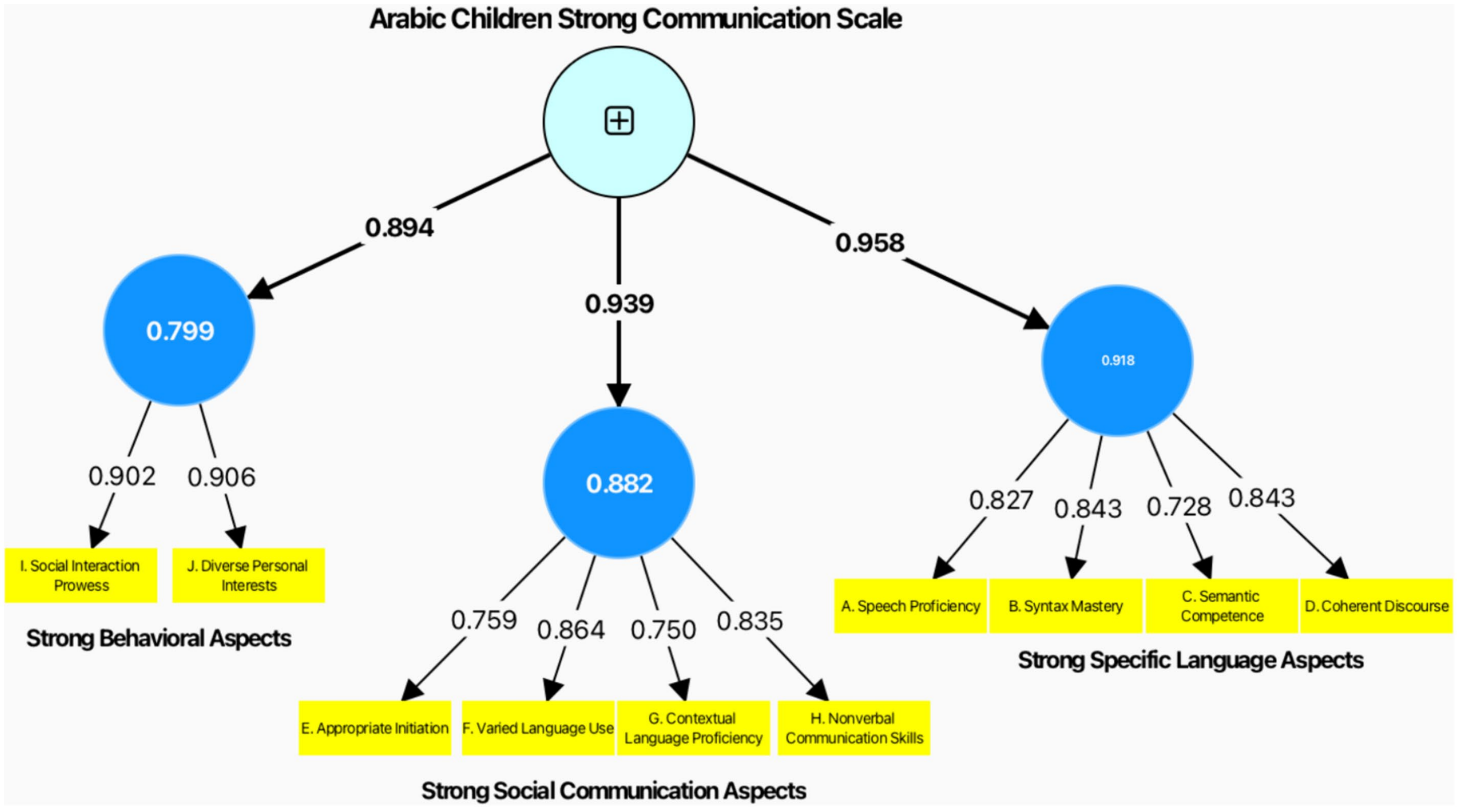
CCA of ACSCS.

Consistent with a systematic methodological approach, CCA is employed to evaluate model performance within partial least squares structural equation modeling (PLS-SEM). Within our specified reflective measurement model (Figure 1), the indicators or items are influenced by the underlying latent variable, which in this case is termed the Arabic children strong communication scale.

To evaluate the construct’s reliability, researchers employed both Cronbach’s alpha and composite reliability. Composite reliability emerged as the more accurate choice due to its weighted nature, as opposed to the unweighted Cronbach’s alpha. Data were analyzed using SmartPLS software, and the measurement model’s validity was assessed through convergent and discriminant validity measures. Table 3 summarizes the indicator loadings, composite reliability, and average variance extracted (AVE) for this assessment.

**Table 3:** Convergent validity and reliability of the constructs.

Construct	Item	Loading	Cronbach’s alpha	Composite reliability (rho_a)	Composite reliability (rho_c)	AVE
Strong specific language aspects	A. Speech proficiency	0.827	0.826	0.83	0.885	0.659
	B. Syntax mastery	0.843				
	C. Semantic competence	0.728				
	D. Coherent discourse	0.843				
Strong social communication aspects	E. Appropriate initiation	0.759	0.816	0.827	0.879	0.646
	F. Varied language use	0.864				
	G. Contextual language proficiency	0.75				
	H. Nonverbal communication skills	0.835				
Strong behavioral aspects	I. Social interaction prowess	0.902	0.776	0.776	0.899	0.817
	J. Diverse personal interests	0.906				

First, strong specific language aspects construct results show that all items (A. Speech proficiency, B. Syntax mastery, C. Semantic competence, D. Coherent discourse) show high loadings (>0.7), indicating a strong association with the underlying construct. Cronbach’s alpha is at 0.826, this suggests acceptable internal consistency within the construct. Composite reliability (rho_a) is with a value of 0.83, and (rho_c): at 0.885, both exceed the recommended threshold of 0.7, confirming the reliability of the construct. Lastly, the AVE value of 0.659 exceeds the minimum accepted level of 0.5, confirming adequate convergent validity for the construct.

Second, strong social communication aspects construct results show that all items E through H also exhibit high loadings, with the lowest at 0.75, which is above the acceptable threshold, suggesting that they are good indicators of the construct. The Cronbach’s alpha of 0.816 is indicative of good internal consistency. Composite reliability (rho_a) is at 0.827, and (rho_c) is at 0.879, these values suggest that the construct is reliably measured. Lastly, the AVE value of 0.646 indicates that the construct has sufficient convergent validity.

Third, strong behavioral aspects construct results show that the loadings for items I and J are very high (>0.9), reflecting a very strong relationship with the construct. The Cronbach’s alpha is a slightly lower alpha of 0.776, yet within acceptable limits, suggests internal consistency. The composite reliability (rho_a) is at 0.776, and (rho_c) is at 0.899, indicating high reliability. Lastly, the result of AVE is at 0.817, this is well above the accepted level, showing excellent convergent validity.

The PLS-SEM results for the three constructs within this study show strong indicator loadings and reliability measures. All constructs have Cronbach’s alpha and composite reliability values well above the recommended threshold of 0.7, indicating that the constructs are measured reliably. Additionally, the AVE values are above 0.5, suggesting good convergent validity for each construct. The strong specific language aspects and strong social communication aspects constructs showed similar patterns in their indicators’ loadings and reliability measures, with strong behavioral aspects showing the highest loadings and AVE, indicating a particularly strong relationship between the indicators and the latent construct. It is worth noting that while these results are favorable, it is also essential to assess discriminant validity to ensure that constructs are distinct from each other. Further analysis and cross-validation with different samples could enhance the robustness of these findings.

### Factors loading on the higher construct

The higher construct, ACSCS, has been further validated through the following reliability and validity measures:

Cronbach’s alpha: with a value of 0.925, the scale demonstrates excellent internal consistency, indicating that the items within the scale are highly correlated and measure the same underlying construct effectively.

Composite reliability (rho_a): at 0.929, and (rho_c): at 0.937, both values significantly exceed the threshold of 0.7, reinforcing the reliability of the construct and indicating that the scale is consistent in its measurement across various items.

Average variance extracted (AVE): the AVE value stands at 0.6, surpassing the recommended level of 0.5 for adequate convergent validity. This suggests that more than half of the variance observed in the items is accounted for by the grand construct.

The high Cronbach’s alpha and composite reliability values corroborate the robustness of the Arabic children strong communication scale, ensuring that the scale is a reliable tool for assessing the communication strengths of Arabic children. Furthermore, the strong AVE value denotes that the construct has a good level of convergent validity, indicating that the items grouped under this construct are indeed appropriate for capturing the essence of the construct. These findings validate the structure of the grand construct and reinforce the conclusions drawn from the indicator loadings. ACSCS is not only theoretically sound but also empirically valid, making it a valuable instrument for both researchers and practitioners in the field of language development. Table 4 shows the ten factors loading on the grand construct.

**Table 4:** Factors loading on the higher construct ACSCS.

Factors	ACSCS
A. Speech proficiency	0.783
B. Syntax mastery	0.804
C. Semantic competence	0.711
D. Coherent discourse	0.808
E. Appropriate initiation	0.66
F. Varied language use	0.849
G. Contextual language proficiency	0.693
H. Nonverbal communication skills	0.797
I. Social interaction prowess	0.8
J. Diverse personal interests	0.816

### Discriminant validity of the ACSCS constructs

The following section evaluates the discriminant validity of ACSCS (HOC) and its associated lower-order constructs (LOCs): strong behavioral aspects, strong social communication aspects, and strong specific language aspects. Discriminant validity ensures that each construct is unique and captures different phenomena. It is assessed by examining the cross-loadings of items to confirm that each item loads more strongly on its associated construct than on any other construct. This assessment is crucial to confirm that the constructs are distinct, and measure intended unique aspects.

First, strong specific language aspects (LOC). Items A–D (Speech proficiency, Syntax mastery, Semantic competence, Coherent discourse) demonstrate higher loadings on this construct compared to the other constructs, indicating good discriminant validity. The highest loadings for syntax mastery (0.843) and coherent discourse (0.843) on their intended construct further validate their strong association with specific language aspects.

Second, strong social communication aspects (LOC). Items E and H (appropriate initiation, nonverbal communication skills) show stronger associations with this construct, with loadings of 0.759 and 0.835 respectively, compared to their loadings on other constructs, suggesting distinctiveness and good discriminant validity. However, varied language use (F) exhibits a loading of 0.864 on the strong social communication aspects construct, which is marginally higher than its loading on the grand construct (0.849), indicating a slightly better fit with this LOC.

Third strong behavioral aspects (LOC). Items I (social interaction prowess) and J (diverse personal interests) show the strongest loadings on the strong behavioral aspects construct, with 0.902 and 0.906 respectively, illustrating excellent discriminant validity for these items.

Lastly, ACSCS (HOC). Across all items, while there are significant loadings on the grand construct, the loadings are consistently higher on their respective lower-order constructs, ensuring that each LOC is capturing a dimension distinct from the HOC.

The analysis confirms that the lower-order constructs within the ACSCS are distinct and measure unique aspects of communication as intended. Each LOC demonstrates a strong and unique association with its corresponding items, satisfying the criteria for discriminant validity. This finding is crucial for the construct’s theoretical framework and practical application, ensuring that each aspect of communication is uniquely and accurately captured, allowing for targeted interventions and research within each domain. However, careful attention should be given to the close loadings of some items across constructs, which may require further exploration to ensure the clarity and precision of the constructs. Table 5 illustrates the discriminant validity of the ACSCS (HOC) and its associated lower-order constructs (LOCs).

**Table 5:** Discriminant validity of the ACSCS.

	Arabic children strong communication scale	Strong behavioral aspects	Strong social communication aspects	Strong specific language aspects
A. Speech proficiency	0.783	0.67	0.674	0.827
B. Syntax mastery	0.804	0.639	0.73	0.843
C. Semantic competence	0.711	0.547	0.677	0.728
D. Coherent discourse	0.808	0.784	0.645	0.843
E. Appropriate initiation	0.66	0.466	0.759	0.576
F. Varied language use	0.849	0.737	0.864	0.764
G. Contextual language proficiency	0.693	0.508	0.75	0.639
H. Nonverbal communication skills	0.797	0.68	0.835	0.701
I. Social interaction prowess	0.8	0.902	0.661	0.74
J. Diverse personal interests	0.816	0.906	0.703	0.735

### Evaluation of the higher-order construct in the structural model

First, the VIF analysis across all constructs related to speech, syntax, semantics, and coherence indicates that multicollinearity is not a significant concern in the current dataset. All VIF values are below the threshold of 5, with most being considerably lower, suggesting that the predictor constructs are relatively independent, and the regression estimates are stable. This allows for a high degree of confidence in the structural model evaluation and the subsequent interpretation of the path coefficients derived from it.

This section evaluates the structural model involving the higher-order construct (HOC) Arabic children strong communication scale’ and its associated lower-order constructs (LOCs): strong specific language aspects, strong social communication aspects, and strong behavioral aspects. The structural model assessment includes analysis of the path coefficients (original sample), the mean of the bootstrap samples (sample mean), the standard deviation of the bootstrap samples (STDEV), T statistics (|O/STDEV|), and *p* values for each path. Additionally, the effect size (*f*^2^) for the HOC and the coefficient of determination (*R*^2^) for each LOC are reported.

1. Strong specific language aspects (LOC). The effect size (*f*^2^) for this LOC on the HOC is substantial at 0.918, indicating a high impact. The *R*^2^ value of 0.958 shows that the HOC explains a significant amount of variance in this LOC. The path coefficient is extremely strong at 0.958, with a negligible standard deviation of 0.009, resulting in a very high T statistic of 101.679, indicating a highly significant relationship (*p* value = 0.00).

2. Strong social communication aspects (LOC). With an effect size of 0.882, the impact of this LOC on the HOC is also considerable. The *R*^2^ value is 0.939, demonstrating that a large proportion of the variance in the strong social communication aspects is accounted for by the HOC. The path coefficient of 0.939, a standard deviation of 0.011, and a T statistic of 83.217 confirm a strong and statistically significant relationship (*p* value = 0.00).

3. Strong behavioral aspects (LOC). The effect size (*f*^2^) of 0.799 suggests a slightly lower yet still significant impact on the HOC compared to the other LOCs. The *R*^2^ value is 0.894, indicating that a substantial variance in strong behavioral aspects is explained by the HOC. The path coefficient is robust at 0.894, with a standard deviation of 0.019, resulting in a T statistic of 46.164, which supports a statistically significant relationship (*p* value = 0.00).

The statistical analysis indicates that ACSCS is a strong predictor of each of the three lower-order constructs, with all paths showing highly significant relationships. The effect sizes are substantial, suggesting that interventions aimed at improving the higher-order construct will likely have significant effects on each of the specific language, social communication, and behavioral aspects of communication skills in Arabic children. Given the high *R*^2^ values, the HOC accounts for a large portion of the variance in each LOC, signifying that the model is robust, and the constructs are well-defined and impactful. The results strongly validate the use of ACSCS in research and practice, highlighting its importance in assessing and developing communication competencies. Table 6 summarizes the results the evaluation of the higher-order construct in the structural model.

**Table 6:** Structural model evaluation.

HOC	F2	LOC	R2	Original sample (O) (path coefficient)	Sample mean (M)	Standard deviation (STDEV)	T statistics (|O/STDEV|)	P value
Arabic children strong communication scale	11.173	Strong specific language aspects	0.918	0.958	0.958	0.009	101.679	0.00
	7.492	Strong social communication aspects	0.882	0.939	0.94	0.011	83.217	0.00
	3.977	Strong behavioral aspects	0.799	0.894	0.894	0.019	46.164	0.00

### Correlation matrix of ACSCS constructs

Pearson’s correlation coefficient (Pearson’s *r*) values are used to measure the linear correlation between pairs of items related to communication proficiency. The Pearson correlation coefficient ranges from −1 to +1. A value closer to +1 indicates a strong positive correlation, meaning as one variable increases, the other tends to increase as well. A value closer to −1 indicates a strong negative correlation, where one variable’s increase corresponds with the other’s decrease. A value around 0 indicates no correlation.

The presented correlation matrix outlines the relationships between the constructs of the ACSCS (HOC) and its associated lower-order constructs (LOCs) categorized into strong specific language aspects, strong social communication aspects, and strong behavioral aspects.

1. Strong specific language aspects (LOC). Constructs A–D, encompassing speech proficiency, syntax mastery, semantic competence, and coherent discourse, demonstrate varying degrees of positive correlations with one another. Coherent discourse (D) has a particularly strong relationship with speech proficiency (A), evidenced by a correlation coefficient of 0.709. These correlations suggest that the constructs within the strong specific language aspects are interdependent with each aspect reinforcing the others.

2. Strong social communication aspects (LOC). Constructs E–H, covering appropriate initiation, varied language use, contextual language proficiency, and nonverbal communication skills, show positive correlations among themselves. Varied language use (F) is notably strongly correlated with nonverbal communication skills (H) with a coefficient of 0.656. This indicates that social communication skills are closely knit, where proficiency in one area may imply competence in another.

3. Strong behavioral aspects (LOC). Constructs I and J, social interaction prowess and diverse personal interests, exhibit strong correlations with other constructs, particularly with each other (0.633), which underscores the importance of behavioral aspects in the overall communication ability of Arabic children.

The high correlations within and across the LOCs indicate that these constructs are not only internally consistent but also complement each other, supporting the multifaceted nature of the HOC. The *** signifies that the correlations are statistically significant at the 0.001 level, providing strong confidence in the reliability of these relationships. The inter-construct correlations validate the predictive capacity of the HOC over its LOCs, aligning with prior findings that indicated the HOC as a strong predictor of each LOC. The correlation matrix confirms the interrelated nature of communication constructs within the Arabic children strong communication scale. The statistically significant correlations suggest that these constructs, while distinct, are part of an interconnected framework that collectively contributes to the overall communication proficiency. Table 7 shows the correlation matrix of ACSCS constructs.

**Table 7:** Correlation matrix among the scale factors.

		A	B	C	D	E	F	G	H	I	J
A	Pearson’s *r*	—									
B	Pearson’s *r*	0.53***	—								
C	Pearson’s *r*	0.428***	0.59***	—							
D	Pearson’s *r*	0.709***	0.607***	0.391***	—						
E	Pearson’s *r*	0.48***	0.54***	0.486***	0.369***	—					
F	Pearson’s *r*	0.608***	0.621***	0.589***	0.66***	0.542***	—				
G	Pearson’s *r*	0.562***	0.527***	0.484***	0.499***	0.451***	0.521***	—			
H	Pearson’s *r*	0.513***	0.651***	0.605***	0.515***	0.508***	0.656***	0.478***	—		
I	Pearson’s *r*	0.547***	0.613***	0.484***	0.747***	0.385***	0.666***	0.42***	0.612***	—	
J	Pearson’s *r*	0.663***	0.542***	0.505***	0.671***	0.456***	0.665***	0.497***	0.616***	0.633***	—

*p < 0.05, **p < 0.01, and ***p < 0.001. A. Speech proficiency, B. Syntax mastery, C. Semantic competence, D. Coherent discourse, E. Appropriate initiation, F. Varied language use, G. Contextual language proficiency, H. Nonverbal communication skills, I. Social interaction prowess, J. Diverse personal interests.

## Discussion

The principal objective of this study was to validate the ACSCS and to evaluate its efficacy in quantifying communication strengths and weaknesses among Arabic-speaking children. This was achieved through the adaptation of the well-established CCC-2, integrating comprehensive theoretical models, and employing rigorous reliability and validity scales and measures. Through the application of EFA and CCA, we sought to rigorously establish the structural validity of the ACSCS.

The detailed analysis, based on the responses from 102 participants, confirmed the ACSCS as a robust and reliable tool for assessing the nuances of communication capabilities among Arabic-speaking children. This validation supports the findings from previous research which demonstrated the effectiveness of the CCC and CCC-2 in assessing diverse linguistic contexts. Such contexts include studies across various languages such as English (Bishop and Baird, 2001), Dutch (Verté et al., 2006), German (Sarimski, 2006), Norwegian (Helland and Heimann, 2007), Spanish (Hoffmann et al., 2013), and Japanese (Tanaka et al., 2017), thereby underscoring the cross-cultural applicability of the CCC frameworks.

The EFA conducted as part of our study not only reinforced the ACSCS’s capability to differentiate between distinct communication strengths and weaknesses but also highlighted its potential as a culturally adapted tool that is sensitive to the specific needs of Arabic-speaking populations. The Cronbach’s alpha values, ranging from 0.776 to 0.860 across various communication dimensions such as strong specific language aspects, strong social communication, and strong behavioral aspects, indicate a high level of internal consistency and reliability of the scale. These values are indicative of the scale’s comprehensive ability to capture coherent and consistent responses, paralleling the reliability observed in the Norwegian adaptation of the CCC-2 used by Helland and Heimann (2007) to distinguish between typically developing children and those referred to psychiatric services due to language impairments.

The ACSCS’s validation introduces it as a highly practical and reliable instrument specifically designed for Arabic-speaking children. It demonstrates significant promise for widespread application in clinical settings throughout the Arab region, reflecting the success of similar tools in identifying and addressing communication disorders in children with conditions like ADHD and traumatic brain injuries as shown in studies by Aghaz et al. (2022), De La Torre Carril et al. (2021), Fisher et al. (2022), Girimaji et al. (2023), and Nowell et al. (2022).

Furthermore, the strong correlations observed within and across the various constructs of the ACSCS during our structural modeling evaluation and correlation matrix analysis provide empirical support for the scale’s integrated approach to assessing communication skills. This interconnectedness enhances the tool’s utility as a comprehensive instrument capable of measuring a spectrum of strengths and weaknesses across linguistic, social, and behavioral dimensions. This holistic approach aligns with the methodologies utilized by Bishop’s CCC-2, enabling a generalized screening of communication disorders and pragmatic and social interaction deficits. The results from this study not only affirm the structural and functional validity of the ACSCS but also highlight its potential as a transformative tool for advancing the understanding and support of communication development in Arabic-speaking children. It sets a precedent for future research aimed at enhancing and refining communication assessment tools within varied linguistic and cultural landscapes.

### Implications for practice

The validation of the ACSCS has multifaceted implications for clinical, educational, and research practices within the Arabic-speaking context. Clinically, the ACSCS serves as a refined instrument enabling practitioners to diagnose and delineate specific communication strengths and weaknesses in children, thereby facilitating individualized intervention strategies. In educational settings, the scale provides educators with a nuanced understanding of each child’s communicative profile, which can inform tailored instructional approaches and support inclusive education initiatives. For researchers, the ACSCS presents an empirically validated tool, opening avenues for future studies exploring the developmental trajectories of communication skills in Arabic-speaking populations. Additionally, the scale’s comprehensive scope, encompassing both strengths and weaknesses across linguistic, social, and behavioral dimensions, allows for a holistic assessment that aligns with contemporary, multifaceted educational and psychological frameworks.

### Limitations

While the ACSCS demonstrates strong psychometric properties, certain limitations warrant consideration. The cross-sectional design of this study precludes the observation of communication development over time, which could be addressed in longitudinal studies. Furthermore, the reliance on convenience sampling and the use of electronic distribution of questionnaires may introduce bias, potentially affecting the generalizability of the findings. The inherent subjectivity of self-reported measures also suggests the need for future studies to corroborate these findings with objective assessment tools. Additionally, the cultural heterogeneity within the Arabic-speaking world necessitates further validation studies across different Arabic dialects and socio-cultural backgrounds to ensure the scale’s broad applicability.

## Conclusion

The ACSCS has demonstrated substantial reliability and validity in assessing communication strengths and weaknesses among Arabic-speaking children. This study has successfully adapted and validated the ACSCS, utilizing robust methodologies, including the EFA and CCA, to confirm its structural integrity and applicative value within the Arabic cultural context. The findings affirm the ACSCS’s effectiveness as a nuanced tool capable of capturing a wide range of communication abilities. The scale’s ability to delineate between different communication competencies highlights its sensitivity to the diverse linguistic features and social nuances characteristic of Arabic-speaking populations. By maintaining high Cronbach’s alpha values across various communication dimensions, the ACSCS proves itself as a consistent and reliable instrument, paralleling the effectiveness of the original CCC-2 and its adaptations in other languages and settings.

The ACSCS’s comprehensive approach allows it to act not only as a diagnostic tool but also as a mechanism for ongoing monitoring of communication development. It facilitates targeted interventions by educators and clinicians, thereby supporting the educational success and social integration of children with communication challenges. The study’s cross-cultural adaptation process also underscores the importance of culturally sensitive assessment tools, which are crucial for accurately reflecting and addressing the needs of diverse populations. Furthermore, the ACSCS contributes to the existing literature by extending the scope of communication assessment tools to Arabic-speaking children, a demographic previously underrepresented in such research. The positive correlations found within and across the ACSCS’s constructs during our analyses reinforce the interconnected nature of communication skills and validate the scale’s structure and theoretical framework.

In summary, the validation of the ACSCS enriches the toolkit available to practitioners and researchers concerned with child development, offering a reliable and culturally appropriate resource for assessing and supporting communication development in Arabic-speaking children. Future research should aim to expand the application of the ACSCS across different Arabic dialects and more diverse socio-economic backgrounds to further enhance its utility and impact.

## Data Availability

The raw data supporting the conclusions of this article will be made available by the authors, without undue reservation.

## Appendix A

**Table A1:** Arabic children’s strong communication scale items.

Items	Construct
يتحدث بوضوح بحيث تكون الكلمات مفهومة بسهولة من قبل شخص لا يعرفه جيدًا.	الكلام الخطاب
يتفاعل بشكل إيجابي عند اقتراح نشاط جديد وغير مألوف.	الاهتمامات
يتحدث بوضوح عما يخطط أو ينوي القيام به في المستقبل (على سبيل المثال ما سيفعله غدًا، أو ينوي فعله في نهاية اجازة الاسبوع).	الترابط المنطقي
يقدر الفكاهة التي تعبر عنها استعارة تهكمية فمثلا سيكون مسروراً بدلا من أن يكون حائر إذا قال شخص ما “أليس الطقس رائع!” هو شديد الحرارة.	استخدام سياق الكلام
ينطق جمل طويلة ومعقدة مثل: “عِنْدَمَا ذَهَبْنَا إِلَى مُبَارَاةِ الْكُرَةِ الْقَدَمِ، رَأَيْتُ إِنْجِلْتَرَا تَفُوزُ”.	النحو القواعد
يُحْسِنُ اسْتِخْدَامَ الإِيمَاءَاتِ أو الاشارات باليد لِتَبْلِيغِ مَعْنَاهُ.	التواصل غير اللفظي
يُظْهِرُ الْقَلَقَ عِنْدَ حزن الاخرين من حوله.	العلاقات الاجتماعية
يَتَكَلَّمُ بِطَرِيقَةٍ سَلِسَةٍ وَوَاضِحَةٍ، ناطقاً جَمِيعَ أَصْوَاتِ الْكَلَامِ بِدِقَّةٍ وَبِدُونِ أَيِّ تَرَدُّدٍ.	الخطاب الكلام
يحافظ على الهدوء في المواقف التي يحاول فيها شخص آخر التحدث أو التركيز (على سبيل المثال عندما يشاهد شخص آخر التلفزيون، أو خلال المناسبات الرسمية مثل الاجتماع أو المناسبة الدينية).	بداية غير مناسبة
يدرك الحاجة إلى أن يكون مهذبا - فمثلا من شأنه أن يتظاهر بالرضا إذا تم إعطاؤه هدية لم تعجبه حقًا، ويتجنب الإدلاء بتعليقات شخصية عن الغرباء.	استخدام سياق الكلام
يَقُومُ بِتَوْفِيرِ مَعْلُومَاتٍ كَافِيَةٍ عِنْدَ الْجَوَابِ عَلَى سُؤَالٍ دُونَ أَنْ يَكُونَ مُتَطَرِّفًا فِي الدِّقَّةِ.	لغة مبتذلة نمطية
يمكنك إجراء محادثة ممتعة ومثيرة للاهتمام معه.	لغة مبتذلة نمطية
يظهر مرونة في التكيف مع المواقف غير المتوقعة: على سبيل المثال لا ينزعج إذا كان يخطط للعب على الكمبيوتر، ولكن عليه أن يفعل شيئًا آخر لأن الحاسوب لا يعمل.	الاهتمامات
يستخدم كلمات مجردة تشير إلى مفاهيم عامة بدلاً من شيء يمكنك رؤيته - على سبيل المثال “المعرفة”، “السياسة”، “الشجاعة”.	علم الدلالة
يبتسم بشكل مناسب عند التحدث إلى الناس.	التواصل غير اللفظي
يستخدم الكلمات التي تشير إلى فئات كاملة من الكائنات، بدلاً من عنصر معين. على سبيل المثال، يشير إلى طاولة وكرسي وأدراج باسم “الأثاث”، أو إلى التفاح والموز والكمثرى باسم “الفاكهة.”	علم الدلالة
يتحدث عن أصدقائه ويظهر الاهتمام بما يفعلونه ويقولونه.	الاجتماعية العلاقات
يشرح حدثًا سابقًا (على سبيل المثال ما فعله في المدرسة، أو ما حدث في مباراة كرة قدم) بوضوح.	الترابط المنطقي
ينتج أو يستخدم جمل تحتوي على اداة الربط “لأن” مثل “أحمد كان لديه كعكة لأنه كان نجح في الامتحان”.	النحو القواعد
يتحدث إلى الآخرين حول اهتماماتهم، بدلاً من اهتماماته الخاصة.	بداية غير مناسبة
